# Associations between formal social support in early postpartum, parenting self-efficacy, and parent-infant bonding: A cross-sectional survey

**DOI:** 10.1016/j.ijnsa.2026.100508

**Published:** 2026-02-11

**Authors:** Elisabeth Schobinger, Alain Lacroix, Anne-Sylvie Ramelet, Antje Horsch

**Affiliations:** a Institute of Higher Education and Research in Healthcare (IUFRS), University of Lausanne, Lausanne Switzerland; b HESAV School of Health Sciences- Vaud, HES-SO University of Applied Sciences and Arts Western Switzerland, Switzerland; c Department Woman-Mother-Child, Lausanne University Hospital, Switzerland

**Keywords:** Mothers, Social support, Early postpartum, Parenting self-efficacy, Fathers, Bonding

## Abstract

**Background:**

Formal social support from healthcare professionals is critical in facilitating the transition to parenthood and may contribute to parental self-efficacy. However, its association with parent-infant bonding remains unclear, and evidence to guide clinical practice is scarce, especially during the postpartum hospital stay.

**Objectives:**

This study aimed to (a) explore associations between formal social support during the postpartum stay, parenting self-efficacy, and parent-infant bonding; and (b) investigate the role of depressive symptoms and other confounding factors.

**Design and Setting:**

A cross-sectional survey was conducted from April 2023 to August 2024 in five hospitals in French-speaking Switzerland.

**Participants:**

420 first-time parents (298 mothers and 122 partners).

**Methods:**

First-time mothers and co-parents completed a cross-sectional survey. Variables were measured using the Hospital Anxiety and Depression Scale, the Birth Satisfaction Scale-Revised, the FOCUS-Postpartum Scale, the Perinatal Infant Care Social Support Scale, the Parenting Expectations Survey, and the Mother-Infant Bonding Scale. Descriptive statistics, correlation analyses, and regression analyses were performed.

**Results:**

A total of 298 mothers and 122 partners answered the survey. Parents reported low satisfaction with the information received during the postpartum hospital stay. In bivariate analyses, formal social support was positively associated with maternal self-efficacy (rho = 0.18, *p* = 0.004) but not with partners’ self-efficacy (rho= -0.07, *p* = 0.45) or parent-infant bonding. After adjustment for confounding factors, the association between formal social support and maternal self-efficacy was no longer significant. In the maternal regression model, satisfaction with informal general support was significantly associated with maternal self-efficacy (β = 0.62, *p* = 0.002). Non-exclusive breastfeeding was associated with higher maternal bonding difficulties (β = 0.34, *p =* 0.008). Partners with a secondary education level reported lower parenting self-efficacy (β = -35.49, *p =* 0.005) and lower bonding quality (β = 0.7, *p =* 0.019) than those with an apprenticeship education. Depressive symptoms were negatively associated with parental self-efficacy (mothers β = -2.44, *p* = 0.015; partners β = -4.57, *p* < 0.001) and bonding (mothers β = 0.08, *p* < 0.001; partners β = 0.07, *p* = 0.002).

**Conclusions:**

Higher satisfaction with informal support was linked to maternal self-efficacy, while depressive symptoms consistently undermined both parental self-efficacy and bonding for both parents. Together with parents’ low satisfaction with information received, these findings underscore the need for tailored support and the importance of clear and consistent communication from healthcare professionals for both mothers and partners.

**What is already known**

• Formal social support is crucial during the early postpartum period.
• Evidence linking formal support to parenting self-efficacy and parent-infant bonding is limited.

Alt-text: Unlabelled box dummy alt text

**What this paper adds**

• Provides new evidence from the very early postpartum period, including both mothers and partners.
• Distinguishes the respective roles of formal and informal social support in relation to parental self-efficacy and bonding.
• Emphasises the role of depressive symptoms and contextual factors in parental self-efficacy and parent–infant bonding.

Alt-text: Unlabelled box dummy alt text

## 1 Background

Becoming a parent for the first time marks a significant life transition, leading to notable social and emotional adjustments (Finlayson et al., 2020; Schobinger et al., 2022). The early postpartum, lasting from the second to the seventh day after birth, is critical for developing parenting confidence (World Health Organisation, 2010). During this period, first-time parents are at higher risk of acute stress, difficulties with infant bonding, and potential mental health complications (Schobinger et al., 2020), and report lower parenting self-efficacy than multiparous parents (Liyana Amin et al., 2018; Samdan et al., 2022), possibly due to the absence of enactive mastery experience –prior successful experiences in infant care– which is key role for self-efficacy (Saether et al., 2023).

Parenting self-efficacy and bonding are central indicators of parental adjustment (Zheng and Gao, 2023; de Waal et al., 2025). Parenting self-efficacy refers to the belief parents hold in their capacity to care for their infant (de Montigny and Lacharité, 2005; Zheng and Gao, 2023). Four primary sources contribute to the development of self-efficacy beliefs: 1) past success experiences; 2) indirect experiences through observing others; 3) verbal encouragement; and 4) physiological and affective states, where positive emotions boost self-efficacy and negative emotions diminish it (Bandura, 1997; Fierloos et al., 2023). Early postpartum period is also when the parent-infant bond forms, encompassing emotional involvement and internal feelings toward the infant (Nakić Radoš et al., 2024; Toivo et al., 2023). Supporting parenting self-efficacy and bonding in the early postpartum may therefore foster a positive transition to parenthood.

Social support is an important protective factor during this transition. Social support is a dynamic, reciprocal process that enables individuals to navigate difficult or stressful situations more effectively, thereby improving the health and well-being of the recipient (Langford et al., 1997; Ekström-Bergström et al., 2022). It can be informal (support from family and close ones) or formal (support from healthcare professionals) (Leahy-Warren et al., 2019). Whilst the benefits of informal support are well described in the literature, evidence regarding formal support is limited, despite its importance during early postpartum. Formal support differs in nature and purpose from informal support. Informal support, typically provided by family and friends, is influenced by the nature of the relational and emotional relationship, whereas formal support from healthcare professionals is considered an intervention to support person-and family-centred care (Voorhees, 2023; Leahy-Warren, 2014). Professional mainly provide informational and appraisal support (e.g., guidance on infant care, reassurance), while informal support is mostly emotional or instrumental (Leahy-Warren, 2014, Francis Xavier et al., 2024). First-time parents often identify their partner as their primary source of emotional support (Francis Xavier et al., 2024; Leahy-Warren et al., 2019). However, first-time fathers tend to rely on formal support for expert guidance (Francis Xavier et al., 2024). This distinction highlights the unique role healthcare professionals can play in enhancing parents’ confidence and skills. Supportive behaviours include actions or interpersonal skills demonstrated by healthcare professionals to assist first-time parents in their transition to parenthood (Schobinger et al., 2025b, Francis Xavier et al., 2024). Healthcare professionals may use a wide range of behaviours to help first-time parents, such as providing information on infant care, yet, when given inappropriately (e.g., contradictory information), it can causes unnecessary stress to parents (Schobinger et al., 2024).

Adequate formal support reduces stress and risk of mental health disorders (World Health Organisation, 2022; Finlayson et al., 2020; Baldwin et al., 2019). When parents receive adequate informal support they develop a sense of security, fostering parenting self-efficacy (Fang et al., 2021). Informal social support is correlated with parenting self-efficacy (Fierloos et al., 2023; Zheng et al., 2018). However, only one study investigated if formal social support from professionals was associated with parenting self-efficacy and found no association (Salonen et al., 2009). In a recent scoping review, Sæther et al. (2023) explored first-time parents' experiences related to parental self-efficacy and highlighted the crucial role of professional support. First-time parents valued emotionally responsive and individualised support, trusting relationships, and continuity of care. They appreciated when professionals were respectful, non-judgmental, and took time to listen to their needs and goals. Fathers emphasised the importance of hands-on experience and disliked passive, overly formal instruction (Saether et al., 2023). These findings suggest that formal support may enhance parenting self-efficacy when it meets the specific needs of new parents. Several studies show that informal social support is positively associated with parent-infant bonding (Stuijfzand et al., 2020; Martin and Brock, 2023), but, so far, there is no evidence for formal social support and bonding.

When parents perceive a lack of formal social support or perceive it as inadequate, they are at higher risk of developing mental health problems (Ayers et al., 2019; Cirino and Knapp, 2019), and an increased risk of seeking emergency care for their newborn within the first two weeks after discharge (Barimani et al., 2014). Fathers and other co-parents may also be affected by a lack of formal social support, experiencing negative emotions and psychological distress (Kothari et al., 2022; Eddy et al., 2019). Despite growing recognition of the father's role, most research still focuses on mothers, and paternal experiences remain underexplored. Qualitative work such as Hodgson et al. (2021) has found that first-time fathers often feel “present but invisible” in perinatal services, citing poor communication and a lack of father-specific support. These findings are echoed in a Swiss qualitative study, where first-time fathers reported distinct needs, such as being included in infant care and receiving reassurance (Schobinger et al., 2022). Similarly, a scoping review identified that a non-inclusive hospital environment and healthcare professionals’ inattentiveness to paternal needs were inhibiting factors for first-time fathers’ parenting self-efficacy (Saether et al., 2023). Such findings underscore the importance of including fathers in studies on formal support, as their perceptions and needs may differ from those of mothers and are essential for developing inclusive, equitable care practices.

Beyond social support, psychosocial, obstetrical, and infant-related factors influence parenting self-efficacy and/or parent-infant bonding. Parental mental health and stress impact first-time parents’ parenting self-efficacy (Fang et al., 2021; Saether et al., 2023) and parent-infant bonding (Ngai and Lam, 2023). Obstetrical factors, such as mode of birth can influence birth satisfaction (Falk et al., 2019), which in turn has been associated with parenting self-efficacy (Brand et al., 2025) and bonding quality (Döblin et al., 2023; Nakić Radoš et al., 2024). Caesarean section and early discharge may enhance first-time fathers’ parenting self-efficacy (Saether et al., 2023). Infant factors may challenge parents' confidence and bonding (Azmoude et al., 2015; Nakić Radoš et al., 2024), especially for first-time parents (Saether et al., 2023). Parental education may shape expectations and skills relevant to early caregiving (Donithen and Schoppe-Sullivan, 2022; Fang et al., 2021). Given these findings, accounting for these factors is essential to fully understand the relationship between formal social support, parenting self-efficacy, and bonding.

In summary, formal social support is an important protective factor in the postpartum period, especially for first-time parents. While research on informal or mixed sources of support has found positive associations with parenting self-efficacy and bonding, evidence on formal social support during the postpartum stay remains scarce. The only study specifically examining formal support found no association, highlighting the need to better understand how supportive behaviours from healthcare professionals may contribute to parenting self-efficacy and bonding in the transition to parenthood.

The first objective of this study was thus to investigate relationships between formal social support and maternal self-efficacy and mother-infant bonding, as well as paternal self-efficacy and father-infant bonding. Based on the literature, we hypothesised that (H1) formal social support would be positively associated with perceived parenting self-efficacy, and (H2) negatively associated with parent-infant bonding difficulties.

The second objective was to identify which psychosocial, obstetrical, and infant factors may be associated with these relationships, for mothers and partners separately. We hypothesised that (H3) psychosocial, obstetric, and infant factors would be associated with perceived parental self-efficacy and bonding for mothers and partners.

## 2 Methods

### 2.1 Design

This study is part of the "SUPERSTAR" project, which includes (1) the development and psychometric validation of a new instrument assessing formal social support (Schobinger et al., 2025a- under review), and (2) the present cross-sectional study, which uses the newly developed instrument to investigate associations between formal social support and psychosocial outcomes in mothers and partners. Participants were recruited from postpartum wards of five hospitals in French-speaking Switzerland (one university hospital and four regional hospitals). All public hospitals in the canton of Vaud were invited to participate; four agreed to take part, while one declined. The selection of these hospitals was purposive, aiming to represent the regional population and to facilitate recruitment within a reasonable geographic area for the research team. Recruitment occurred from April 2023 to July 2024. The research team approached participants during their postpartum stay, provided study information, and offered a leaflet with key information and a QR code linking to an anonymous online survey. To foster recruitment leaflets about the study were placed in the waiting rooms of gynaecologists and midwives and social media (LinkedIn, Instagram, and Facebook) was also used to promote the study.

### 2.2 Participants and setting

Participants were invited to complete the questionnaire within the first month postpartum if they were first-time parents (over 18 years of age), gave birth to a healthy full-term newborn (≥37 gestational weeks), and had been present during birth (for partners). Furthermore, they needed to have been present during the postpartum stay at the hospital. Partners were considered present during the postpartum stay if they stayed overnight or were present at any time during the day at the hospital. Exclusion criteria included parents who were non-French speakers, whose infants were admitted to the Neonatal Intensive Care Unit (NICU), or experienced stillbirth, or whose infant died within 24 h post-delivery, as the study focused on a low-risk population.

Partners were invited to participate if present during birth and the postpartum stay. To maximise inclusion, midwives were asked whether the partner was a first-time parent, especially in cases where the mother had multiple children, but the partner might be experiencing parenthood for the first time. In such cases, the partner was invited to participate. Conversely, partners who already had children from previous relationships were excluded to maintain focus on first-time parenthood.

In Switzerland, health insurance is mandatory, and 98 % of women give birth in hospitals, with caesarean sections accounting for 33 % of births (Office fédéral de la statistique, 2019). The typical postpartum length of hospital stay is 2 to 3 days after a vaginal birth and 3–4 days after a caesarean section. Parents receive written information on self-care, newborn care and community resources, including midwifery services. Mothers and newborns are entitled to 16 postpartum midwife visits within the first eight weeks.

### 2.3 Sample size

Sample size was based on the findings of previous studies reporting medium-sized correlations between informal social support and parenting self-efficacy (Leahy-Warren et al., 2012; Shorey et al., 2014) and small correlations with parental bonding (Stuijfzand et al., 2020). G*Power analysis for bivariate correlation (two-tailed), assuming a small-to-moderate effect size (*r* = 0.20, α = 0.05) and 0.80 power, estimated a sample of 200 mothers and 200 partners.

For multiple regression testing H3 (moderate effect size (*f*² = 0.15, α = 0.05), power = 0.80, 20 predictors) 160 participants were required. The larger bivariate correlation estimate was retained as the final target sample. Adding 15 % for potential participant dropout and questionnaires with no usable data, the total target sample size was 460 parents (230 mothers, 230 partners).

### 2.4 Data collection

Participants completed an online anonymous survey on their experience of formal social support received during their postpartum hospital stay. Parents were invited to complete the survey at any time within the first month postpartum to allow flexibility. Mothers and partners participated individually; with couple participation encouraged. The completion time of the survey was between 20 and 30 min.

### 2.5 Instruments

Sociodemographic and birth-related variables included gestity, complications during pregnancy and in the immediate postpartum, mode of delivery, infant’s birth weight and gestational age, infant feeding at discharge, and postpartum duration stay.

#### 2.5.1 Social support received from healthcare professionals

Formal social support parents received during the postpartum stay was measured using the 35-item FOCUS-Postpartum Scale, developed and validated with postpartum women. Participants rated healthcare professionals’ social support behaviours from 0 (*did not meet their needs at all*) to 4 (*completely met their needs*), providing a possible total score ranging from 0 to 140, where higher scores indicate a greater extent to which parents’ support needs were met. It includes an overall factor and four dimensions: “optimising parents’ learning” (13 items, ranging 0 to 52), “providing respectful care” (12 items, ranging 0 to 48), “caring with empathy” (5 items, ranging 0 to 20), and “admitting and orientating parents” (5 items, ranging 0 to 20) (Schobinger et al., 2025a- under review). The scale was developed following a rigorous multi-phase process aligned with COSMIN guidelines. Item generation was based on a literature review and qualitative interviews with postpartum mothers and fathers, followed by item selection and evaluation through a Delphi survey involving 90 first-time parents. The item pool was further refined with input from perinatal experts and parents. The scale demonstrated strong internal consistency in the initial validation study with postpartum mothers with α = 0.97, and its structural validity was supported by exploratory and confirmatory factor analyses (Schobinger et al., 2025a- under review). In the current study sample including both mothers and fathers/partners, the scale showed excellent reliability, with α = 0.97.

#### 2.5.2 Social support in the perinatal period

Social support from healthcare professionals and informal support from family was measured using the Perinatal Infant Care Social Support instrument, a validated measure of postpartum social support for mothers (Leahy-Warren et al., 2019). Functional support is measured using 19 items rated on a 4-point Likert scale. This questionnaire was translated into French and culturally adapted according to Wild et al. (2005), with α = 0.92 in this study.

#### 2.5.3 Satisfaction with formal and informal social support

Satisfaction with formal social support was assessed using five questions on a 100-mm visual analogue scale, ranging from 0 (*extremely dissatisfied*) to 100 (*extremely satisfied*) (Voutilainen et al., 2016). One question measured overall satisfaction, while the others addressed informational, instrumental, appraisal, and emotional support. Internal consistency for the five items was excellent (α = 0.93).

Exploratory factor analysis supported the structural validity of the scale. The suitability of the data for factor analysis was confirmed by a Kaiser-Meyer-Olkin measure of sampling adequacy of 0.867. Bartlett’s test of sphericity was significant (χ² = 1691.27, df = 10, *p* < 0.001), and the determinant of the correlation matrix (0.010) indicated no issue of multicollinearity. The exploratory factor analysis revealed a unidimensional structure: (first factor eigenvalue = 3.78, explaining 76 % of the variance, all loadings > 0.85). Although a composite score was possible, each item represents a distinct theoretical dimension of formal support. Therefore, we chose to analyse them individually to reflect the nuanced aspects of parents’ satisfaction. Scores were categorised as “*not satisfied*” (<50), “*moderately satisfied*” (50–79) and “*satisfied*.” (≥80), aligning with previous research, where ≥80 % is commonly interpreted as “*satisfied or very satisfied*” (Beshir et al., 2022; Vogel et al., 2019).

Satisfaction with informal social support was measured using a response format similar to formal support (five questions, each representing a distinct aspect of support). Exploratory analyses supported a single-factor structure (first factor eigenvalue = 2.52; loadings ranging from 0.46 to 0.79), and good internal consistency (α = 0.81).

#### 2.5.4 General psychological distress

General psychological distress of parents was assessed using French version of the Hospital Anxiety and Depression Scale (Boini and Langevin, 2020). Anxiety and depression were measured with the subscales. The 14 self-reported items are rated on a 4-point Likert scale, with higher score indicating greater severity (range from 0 to 21 for each dimension) (Zigmond and Snaith, 1983). The Hospital Anxiety Depression Scale has good psychometric properties (Zigmond and Snaith, 1983). For this study, α was 0.84 for general distress, 0.76 for anxiety, and 0.78 for depression.

#### 2.5.5 Birth satisfaction

Subjective birth experience was assessed using the 10-item Birth Satisfaction Scale-Revised, which measures postpartum birth satisfaction in mothers (Hollins Martin and Martin, 2014). Items are rated on a 4-point Likert scale; total scores range from 0 to 40, with a higher score indicating greater satisfaction. The scale has a good internal consistency (α = 0.79) and has been translated and validated in French (Devita et al., 2026). In our study, internal consistency was α = 0.75.

#### 2.5.6 Perceived parental self-efficacy

The French version of the Parenting Expectations Survey was used to assess parenting self-efficacy in the early postpartum (de Montigny, 2002). This 25-item self-report scale, with responses rated on a 10-point Likert scale (total score range 0 to 250), has been validated in French for women at one month postpartum (de Montigny and Lacharité, 2002; Razurel et al., 2017). In this study α = 0.92 for mothers and α = 0.88 for partners.

#### 2.5.7 Parent-infant bonding

The 8-item Mother-to-Infant Bonding scale assesses maternal feelings and the bonding quality on a 4-point Likert scale, with higher scores indicating problematic bonding (Taylor et al., 2005). It has fair reliability (α = 0.71), was validated in French for mothers (Bienfait et al., 2017), and used with both parents in a Swiss study (α = 0.77) (Horsch et al., 2017). In this study, Cronbach’s α was 0.67.

### 2.6 Data analysis

We used *t*-tests to compare sociodemographic differences between mothers and partners, and logistic regression to assess differences between complete and incomplete responses. Due to non-normal distribution of some continuous variables (e.g., support received, satisfaction, and bonding scores), differences between mothers and partners were assessed using the Wilcoxon rank-sum test (Mann–Whitney U test). For categorical variables (e.g., satisfaction yes/no), group differences were analysed using chi-square tests or Fisher’s exact tests when cell counts were small. Although not directly related to the main hypotheses, these comparative analyses aimed to provide a more detailed characterisation of the sample and to assess potential response bias, thereby supporting the internal validity and generalisability of the findings and are thus succinctly presented in the results section.

To test hypothesis one (H1) and two (H2), Spearman correlations coefficients were calculated between formal social support and parenting self-efficacy and bonding, due to non-normal distributions. Associations between FOCUS-Postpartum scores and Parenting Expectations Survey, as well as between FOCUS-Postpartum scores and Mother-Infant Bonding Scale, were analysed using a linear regression, adjusting for sociodemographic, obstetric, and infant-related factors, testing for hypothesis three (H3). These are referred to as the “parenting self-efficacy model” and the “bonding model” for mothers and partners, respectively. Covariates were selected a priori based on the literature and their relevance for each outcome. Regression assumptions (linearity, independence, homoscedasticity, normality of residuals) and variance inflation factors were examined. The main variable of interest in this study was the level of formal support received from healthcare professionals, measured with the FOCUS-postpartum scale. Satisfaction with formal social support was also assessed; however, it was highly correlated with the formal support score (overall satisfaction rho=0.72, *p* < 0.001; satisfaction with formal informational support rho=0.76, *p* < 0.001; satisfaction with formal instrumental support rho=0.79, *p* < 0.001; satisfaction with formal appraisal support rho=0.75, *p* < 0.001; satisfaction with formal emotional support rho=0.69, *p* < 0.001). After removing satisfaction with formal social support variables, variance inflation factors for other variables were below commonly accepted thresholds, indicating no problematic multicollinearity. Breusch–Pagan test indicated heteroskedasticity for mothers’ parenting self-efficacy model. Consequently, estimates for this model were reported with robust standard errors. For the parenting self-efficacy model in partners, all assumptions were met. Although satisfaction with informal and formal social support was categorised for descriptive purposes (to report the proportion of participants who were satisfied, moderately satisfied, or not satisfied), it was treated as a continuous variable in the regression analyses.

As the parent-infant bonding scores were zero-inflated (mothers: 36.3 %; partners: 31.9 %) and right-skewed, consistent with a mixture of “no bonding difficulties” (zeros) and “bonding difficulties” (positive values), we employed a hurdle-gamma regression approach combining: a logistic component (modelling zero vs. non-zero responses), which was specified as intercept-only and a Gamma General Linear Model (modelling the truncated positive values, accounting for skewness). Whilst the logistic component was included to account for the data structure, our primary analytical focus was on the Gamma Generalised Linear Model, which provided interpretable estimates of associations with bonding difficulties. The logistic model results are not reported, as they did not yield additional insights relevant to the study objectives.

Model diagnostics were performed using the Diagnostics for Hierarchical Regression Models package, which provides simulation-based residual checks for non-Gaussian models, including tests for uniformity and dispersion. To evaluate the robustness of the primary Gamma Generalised Linear Model results, sensitivity analyses were conducted by fitting alternative models (e.g., Tweedie, Negative Binomial, Log-normal) to the subset of positive Mother-Infant Bonding scores. Model performance was compared using the Akaike Information Criterion and simulation-based diagnostics for residual uniformity and dispersion, implemented via the Diagnostics for Hierarchical Regression Models package. Lower Akaike Information Criterion values indicate better overall fit, while uniformity and dispersion tests assess the adequacy of residual distribution and variance structure (ideal: small D and large *p*-value for uniformity; dispersion close to 1 with large *p*-value). These comparisons allowed us to determine whether alternative specifications provided a substantially better fit than the Gamma Generalised Linear Model. The Gamma Generalised Linear Model remained the most parsimonious and theoretically justified model across comparisons (see Table 1). A Log-normal model was also fitted but excluded due to theoretical mismatches with the data’s skewness.

**Table 1:** Sensitivity analysis results.

Participants	Model	Akaike Information Criteria	Uniformity	Dispersion
Mothers	Gamma	455.92	*D* = 0.099, *p* = 0.146	φ=1.221, *p* = 0.256
	Tweedie	460.68	*D* = 0.102, *p* = 0.126	φ=1.043, *p* = 0.624
	Negative binomial	508.98	*D* = 0.09, *p* = 0.223	φ=1.031, *p* = 0.76
Partners	Gamma	229.6	*D* = 0.105, *p* = 0.496	φ=0.93, *p* = 0.968
	Tweedie	237.14	*D* = 0.093, *p* = 0.65	φ=0.614, *p* = 0.184
	Negative binomial	249.98	*D* = 0.136, *p* = 0.176	φ=0.486, *p* < 0.001

Finally, statistical power for all regression models (parenting self-efficacy and bonding models for mothers and partners) was estimated using bootstrap resampling based on the observed effect sizes (5000 iterations). Pairwise comparisons between all levels of categorical predictors (e.g., education, mode of birth) were conducted to provide additional descriptive detail and to examine potential differences across levels of categorical covariates, using post hoc tests with Bonferroni correction to control the family-wise error rate. Effect sizes were reported as mean differences (95 % confidence intervals) between adjusted group means, along with adjusted *p*-values. All statistical analyses were performed using R (R Core Team, 2024) and Stata version 18 (StataCorp LLC, College Station, TX, USA). A two-tailed *p*-value of < 0.05 was considered statistically significant.

Mothers and partners were analysed separately to account for their distinct experiences of formal support during postpartum hospitalisation, and the individual-level nature of parenting self-efficacy and bonding. This approach avoided assuming equivalence across parents and prevented partners’ data from being overshadowed by the larger maternal sample. Since most participants responded individually rather than as couples, dyadic analyses were not feasible. For descriptive comparisons, participants were treated as independent units, as pairing information was unavailable.

#### 2.6.1 Handling of missing data

Fifty-seven participants who did not respond to any of the items of the Parenting Expectations Survey or the Mother-Infant Bonding Scale were excluded from the analyses. Missing data patterns of these questionnaires were examined using Little’s missing completely at random test. For the Parenting Expectations Survey, data were missing completely at random (χ²(737) = 774.58, *p* = 0.164), with 1.3 % missing data in the remaining dataset. For the Mother-Infant Bonding Scale, missing completely at random assumption was violated (χ²(34) = 88.3, *p* < 0.001), with 0.3 % missing data remaining. Little's missing completely at random tests were performed separately for Parenting Expectations Survey and the Mother-Infant Bonding Scale as they measured distinct constructs with potentially different missingness mechanisms and as these were the only questionnaires with missing data requiring imputation. For completeness, a pooled test of all imputed variables from both questionnaires confirmed the data were missing completely at random, (χ²(1073) = 1032.56, *p* = 0.808), with 36 missingness patterns. Given the very low level of missingness and absence of systematic bias, missing values for both questionnaires were imputed using predictive mean matching method from the mice package. This method is under the missing at random assumption, which preserves the original data distribution, handles ordinal data well, and reduces the risk of introducing bias compared to other imputation methods (Van Buuren, 2018). Five imputed datasets were initially generated; however, the imputation was performed solely to obtain complete questionnaire scores for these primary outcomes, and only a single completed dataset was used for subsequent analyses. Descriptive statistics pre- and post-imputation were nearly identical (mean difference < 0.1 % of the SD), indicating negligible impact. Given the minimal missingness, formal sensitivity analyses for missing not at random were deemed unnecessary (White et al., 2011; Van Buuren, 2018).

### 2.7 Ethical considerations

This study was approved by the Human Research and Ethics Committee of the Canton of Vaud, Switzerland (Project No 2021-00762). Written informed consent was not necessary, as the study was categorised as anonymous, and completion of the online survey implied consent.

## 3 Results

### 3.1 Sample characteristics

A total of 420 participants (298 mothers (71 %) and 122 (29 %) partners) took part in this study and 363 (256 mothers and 107 partners) completed the parenting self-efficacy and bonding questionnaires. The majority were Swiss (65.6 %), had a university degree (68.6 %), and lived together (75 %). The majority gave birth at term (66.7 %) and in a university hospital (60.9 %). Postpartum complications were mainly haemorrhage (*n* = 17, 58.6 %) and severe perineal tear (*n* = 33, 8 %) (see Table 2).

**Table 2:** Sample characteristics.

	Mothers (*N* = 298)	Partners (*N* = 122)	*P*-value	95 % Confidence interval
Age (years; mean, SD)	32.3 ± 4.6	33.2 ± 4.3	0.06 a	−1.86 0.04
Time elapsed between birth and questionnaire completion (days; mean, SD)	13 ± 7.35	11.31 ± 7.35	0.002 b	−0.31 −0.06
Postpartum length of stay (days; mean, SD)	3.37 ± 1.19	3.27± 1.24	0.46 b	−0.16 0.07
Nationality			0.71 c	
Swiss	185 (64.2 %)	82 (68.9 %)		
French	37 (12.8 %)	19 (16 %)		
Italian	14 (4.9 %)	6 (5 %)		
Portuguese	17 (5.9 %)	2 (1.7 %)		
Belgian	8 (2.8 %)	3 (2.5 %)		
Spanish	8 (2.8 %)	2 (1.7 %)		
Origin			0.18 c	
Europe	267 (90.5 %)	114 (95.8 %)		
Latine America	6 (2 %)	3 (2.5 %)		
Africa	8 (2.7 %)			
North America	4 (1.7 %)	2 (1.7 %)		
Asia	6 (2 %)			
Civil status			0.56 c	
Married/in a relationship	217 (73.3 %)	95 (77.9 %)		
Single	75 (25.3 %)	25 (20.5 %)		
Divorced	4 (1.4 %)	2 (1.6 %)		
Previous pregnancy			0.008 c	
None	233 (78.4 %)	115 (94.3 %)		
One	48 (16.2 %)	6 (4.9 %)		
Two	9 (3 %)	1 (0.8 %)		
Previous perinatal loss			0.18 c	
No	231 (77.8 %)	102 (83.6 %)		
Yes	66 (22.2 %)	20 (16.4 %)		
Prenatal class attendance			0.73 c	
Yes	250 (85.6 %)	106 (86.9 %)		
No	42 (14.4 %)	16 (13.1 %)		
Complication during pregnancy			0.23 c	
No	250 (86.2 %)	98 (81.7 %)		
Yes	40 (13.8 %)	22 (18.3 %)		
Mode of birth			0.95 c	
Spontaneous vaginal birth	162 (55.7 %)	66 (55.5 %)		
Instrumental forceps	32 (11 %)	14 (11.8 %)		
Instrumental vacuum assisted	33 (11.3 %)	16 (13.4 %)		
Planned Caesarean section	22 (7.6 %)	8 (6.7 %)		
Emergency Caesarean section	42 (14.4 %)	15 (12.6 %)		
Postpartum complications			0.01 c	
No	258 (89 %)	96 (80 %)		
Yes	31 (11 %)	24 (20 %)		
Infant sex			0.33 c	
Girl	144 (49.8 %)	65 (55.1 %)		
Boy	145 (50.2 %)	53 (44.9 %)		
Infant feeding at discharge			0.64 c	
Breast	227 (76.2 %)	96 (80 %)		
Bottle	24 (8 %)	7 (5.8 %)		
Both	47 (15.8 %)	17 (14.2 %)		
Gestational age at birth (weeks)	39.63 ± 1.32	39.57 ± 1.42	0.56 b	−0.16 0.09
Birth weight (grams)	3317 ± 436	3304 ± 377	0.77 a	−0.76 103.08
General psychological distress (Median; IQR)^1^	11 (9)	7 (6)		
Anxiety	6 (5)	5 (4)	<0.001 b	−0.49 −0.25
Depression	4 (4.5)	2 (4)	0.0001 b	−0.37 −0.23

1 IQR = Interquartile Range. Mean and SD are reported for normally distributed variables; Median and IQR are reported for non-normally distributed variables.

a t-test.

b : ranksum test .

c : Chi-square test.

Most partners (*n* = 83, 68.6 %) stayed overnight during the postpartum stay.

No differences between participants who completed the Parenting Expectations Survey and bonding questionnaires and those who did not were found.

### 3.2 Formal support received from healthcare professionals and satisfaction

Formal support median scores were 109 (IQR = 40.5) for mothers and 115 (IQR = 32) for partners, with no statistically significant difference (*p* = 0.066).

Most participants were satisfied with the support received. Among mothers, 72 % were satisfied with general formal support and partners’ reported satisfaction was 81.8 %. The detailed categories of satisfaction are shown in Table 3.

**Table 3:** Formal support received and Satisfaction.

	Mothers Median (IQR)	Partners Median (IQR)	*P*-value ranksum test	95 % Confidence Interval
**Support received**				
Optimising parents’ learning	42 (17)	44 (12)	0.29^a^	−0.05 0.19
Providing respectful care	36 (17.5)	39 (13)	0.07^a^	−0.008 0.24
Caring with empathy	18 (5)	19 (3.5)	0.06^a^	−0.001 0.24
Admitting and orientating parents	15 (7)	15 (6)	0.13^a^	−0.24 0.22
**Satisfaction (N (%))**	satisfied mothers	satisfied partners	*P*-value chi2 test	
Satisfaction with general formal support	191 (72.6 %)	90 (81.8 %)	0.16^b^	−3.67 11.06
Satisfaction with formal informational support	152 (57.1 %)	69 (62.1 %)	0.36^b^	- 3.39 7.51
Satisfaction with formal instrumental support	165 (62.1 %)	80 (72.7 %)	0.14^b^	- 3.19 11.77
Satisfaction with formal appraisal support	160 (60.6 %)	72 (65.5 %)	0.62^b^	- 4.38 6.28
Satisfaction with formal emotional support	195 (74.1 %)	91 (81.9 %)	0.26^b^	- 3.75 9.15

IQR: interquartile range: ^a:^ ranksum test ^b:^ chi2 test.

### 3.3 Parenting self-efficacy and bonding

Mothers’ median parenting self-efficacy was 188 (IQR=42) and bonding 1 (IQR=2), while partners’ parenting self-efficacy was 184.5 (IQR=41) and bonding 1 (IQR=3) (see Table 4).

**Table 4:** Description of main outcomes.

	Mothers	Partners	*P*-value ranksum test	95 % Confidence interval
Parenting self-efficacy (median; IQR)	188 ; 42	184.5 ; 41	0.5	−0.17 0.08
Bonding (median; IQR)	1 ; 2	1 ; 3	0.19	−0.04 0.21
Satisfaction of birth experience (median; IQR)	30; 9	30; 9	0.84	−0.14 0.11

IQR: interquartile range.

### 3.4 Associations between formal support and parenting self-efficacy and parent-infant bonding

Formal support was positively associated with maternal parenting self-efficacy (rho = 0.18, *p* = 0.002) but not with partners’ parenting self-efficacy (rho = −0.07, *p* = 0.45). Furthermore, none of the subscales correlated with partners’ parenting self-efficacy.

Formal social support received during the postpartum stay was not associated with mother-infant (rho = −0.002, *p* = 0.97) or father-infant bonding (rho = −0.07, *p* = 0.42). (See Table 5)

**Table 5:** Associations between formal support and parenting self-efficacy and bonding scores.

	Maternal parenting self-efficacy	Mother-infant bonding	Partners’ parenting self-efficacy	Father-infant bonding
Formal support	0.18**	−0.002	−0.07	0.07
Optimising parents’ learning	0.20**	−0.004	−0.07	0.11
Provision of respectful care	0.17**	−0.02	−0.04	0.09
Admitting and orientating parents	0.14*	−0.01	0.06	−0.06
Caring with empathy	0.12	−0.07	−0.16	0.12

Rho values that are in bold are significant: Significance levels: *p <0 .05, **p < 0.01.

### 3.5 Factors associated with maternal parenting self-efficacy and mother infant-bonding

The linear regression model including psychological and sociodemographic covariates explained 47.3 % of the variance in maternal parenting self-efficacy (R^2^ = 0.4730) [X^2^(25) =132.65; *p* < 0.001]. Higher depressive symptoms were significantly associated with lower maternal parenting self-efficacy (β = −2.447, *p* = 0.015), while greater satisfaction with informal support was associated with higher maternal parenting self-efficacy (β = 0.623, *p* = 0.002). Maternal anxiety and satisfaction with instrumental informal support showed marginal associations (see Table 6). Among the significant predictors of maternal parenting self-efficacy, depressive symptoms showed the strongest association.

**Table 6:** Multiple regression model for maternal parenting self-efficacy and Gamma Generalised Linear Model regression for mother infant-bonding.

(*N* = 208)	Coefficient	Robust Std. err	t	*p*	95 %CI
Maternal Parenting self-efficacy a					
Formal social support	0.077	0.077	1.01	0.312	−0.074 0.228
Age	−0.246	0.475	−0.52	0.605	−1.177 0.685
Education					
Secondary	−8.31	6.412	−1.30	0.195	−20.878 4.258
Tertiary	−7.677	5.295	−1.45	0.147	−18.055 2.701
Depressive symptoms	−2.447	1.003	−2.44	0.015	−4.413 −0.481
Anxiety symptoms	−1.566	0.941	−1.66	0.096	−3.41 0.278
Birth preparation class attendance					
No	−1.234	5.419	−0.23	0.82	−11.855 9.387
Complication during pregnancy					
None	1.855	6.158	0.30	0.763	−10.215 13.925
Mode of birth					
Forceps assisted delivery	−0.12	7.439	−0.02	0.987	−14.7 14.46
Vacuum assisted delivery	−7.8821	7.41	−1.06	0.287	−22.406 6.642
Planned caesarean section	10.078	7.061	1.43	0.154	−3.762 23.918
Emergency caesarean section	7.775	4.972	1.56	0.118	−1.97 17.52
Birth satisfaction	0.399	0.343	1.16	0.245	−0.273 1.071
Postpartum complication					
None	3.069	7.78	0.39	0.693	−12.18 18.318
Postpartum length of stay	0.044	1.633	0.03	0.978	−3.157 3.245
Time elapsed between birth and questionnaire completion	0.213	0.289	0.74	0.465	−0.353 0.779
Infant sex					
Boy	−1.989	4.269	−0.47	0.641	−10.356 6.378
Infant birth weight	−4.379	5.995	−0.73	0.465	−16.129 7.371
Feeding mode					
Mixed or formula feeding	6.475	5.736	1.13	0.259	−4.768 17.718
Perinatal Infant Care Social support score	0.586	0.422	1.39	0.165	−0.241 1.413
Satisfaction with informal general support	0.623	0.203	3.07	0.002	0.225 1.021
Satisfaction with informal informational support	0.06	0.105	0.57	0.568	−0.146 0.226
Satisfaction with informal instrumental support	−0.225	0.136	−1.65	0.099	−0.492 0.042
Satisfaction with informal appraisal support	0.153	0.21	0.73	0.466	−0.259 0.565
Satisfaction with informal emotional support	−0.189	0.15	−1.25	0.21	−0.492 . 0.042
**(*N*=134)**	**coefficient**	**Std. error**	**z**	***p***	**95 %CI**
**Mother infant bonding**^b^					
Formal social support	0.003	0.002	1.27	0.203	−0.002 0.008
Age	0.036	0.015	2.44	0.015	0.007 0.065
Education					
Secondary	0.054	0.228	0.24	0.811	−0.392 0.509
Tertiary	0.155	0.168	0.92	0.357	−0.183 0.482
Depressive symptoms	0.083	0.019	4.31	<0.001	0.045 0.122
Anxiety symptoms	0.003	0.018	0.16	0.875	−0.032 0.038
Complication during pregnancy					
None	−0.006	0.154	−0.04	0.969	−0.317 0.292
Mode of birth					
Forceps assisted delivery	−0.072	0.15	−0.48	0.632	−0.364 0.229
Vacuum assisted delivery	−0.12	0.19	−0.63	0.528	−0.485 0.268
Planned caesarean section	−0.292	0.244	−1.20	0.23	−0.758 0.207
Emergency caesarean section	−0.072	0.2	−0.36	0.718	−0.458 0.334
Birth satisfaction	−0.009	0.009	−1.02	0.307	−0.028 0.009
Postpartum complication					
None	−0.018	0.152	−0.12	0.908	−0.328 0.274
Postpartum length of stay	−0.025	0.049	−0.51	0.612	−0.121 0.073
Time elapsed between birth and questionnaire completion	−0.004	0.008	−0.51	0.611	−0.019 0.012
Infant sex					
Boy	0.005	0.114	0.04	0.964	−0.221 0.229
Infant birth weight	0.004	0.13	0.03	0.973	−0.252 0.263
Feeding mode					
Mixed or formula feeding	0.344	0.131	2. 46	0.008	0.09 0.606
Perinatal Infant Care Social support score	−0.009	0.008	−1.07	0.285	−0.024 0.007
Satisfaction with informal general support	0.009	0.005	1.61	0.107	−0.002 0.019
Satisfaction with informal informational support	−0.006	0.003	−1.84	0.066	−0.013 0
Satisfaction with informal instrumental support	−0.003	0.005	−0.69	0.493	−0.012 0.006
Satisfaction with informal appraisal support	0.008	0.004	1.82	0.068	−0.001 0.016
Satisfaction with informal emotional support	−0.005	0.004	−1.35	0.177	−0.013 0.002

a Model fit: Adjusted R² = 0.473, X^2^(25) = 123.648, *p* < 0.001.

b Model fit: R² = 0.521.

In the hurdle-gamma regression model, 52.1 % of the variance in mother-infant bonding scores was explained (see Table 6). Depressive symptoms were associated with greater mother-infant bonding difficulties (β = 0.083, *p* < 0.001), corresponding to an 8.7 % increase in bonding difficulties per unit increase in depressive symptoms. Older maternal age was also associated with more bonding difficulties (β = 0.036, *p* = 0.015). Mothers who exclusively breastfed reported better infant bonding than those using mixed feeding, as indicated by lower Mother-Infant Bonding scores (β = 0.344, *p* = 0.008). Satisfaction with informal informational and appraisal support, were only marginally significant (*p* = 0.066 and 0.068) and underpowered (power = 0.41– 0.48). Among significant predictors, infant feeding mode showed one of the strongest associations with bonding difficulties, with mothers who exclusively breastfed reporting fewer difficulties than those using mixed feeding.

### 3.6 Factors associated with partners’ parenting self-efficacy and father-infant bonding.

The linear regression model including psychological and sociodemographic covariates, yielded a model fit of F(25, 67) = 2.157; *p* = 0.007 (see Table 7) for partners’ parenting self-efficacy. It explained 44.6 % of the variance of partners’ parenting self-efficacy (R^2^ = 0.446). Higher depressive symptoms were significantly associated with lower partners’ perceived self-efficacy (β = −4.569, *p* < 0.001), representing the strongest continuous predictor. Partners with secondary education reported lower self-efficacy compared to those with apprenticeship (β = −35.491, *p* < 0.005) (Table 7). Post-hoc comparisons (Table 8) using estimated marginal means with Bonferroni adjustment showed a significant trend in which partners with “secondary” education had a lower expected mean of parenting-self efficacy score than the “apprenticeship” group (estimated marginal means difference = 35.5, *p =* 0.014). Length of postpartum stay showed a marginal association. Post-hoc power analysis indicated that the observed effect sizes were underpowered in our sample.

**Table 7:** Multiple regression model for partners’ parenting self-efficacy and Gamma Generalised Linear Model regression father-infant bonding.

(*N* = 93)	Coefficient	Std. err	t	*p*	95 % CI	power
**Partners’ parenting self-efficacy**^a^						
Formal social support	−0.151	0.124	−1.21	0.231	−0.394 0.092	0.296
Age	0.374	0.737	0.51	0.614	−1.071 1.819	0.078
Education						
Secondary	−35.491	12.169	−2.92	0.005	−59.342 −11.64	0.704
Tertiary	−10.568	7.578	−1.39	0.168	−25.421 4.285	0.342
Depressive symptoms	−4.569	0.994	−4.60	>0.001	−6.517 −2.621	0.974
Anxiety symptoms	−0.068	1.048	−0.06	0.948	−2.122 1.986	0.116
Birth preparation class attendance						
No	9.702	9.046	1.07	0.287	−8.028 27.432	0.144
Complication during pregnancy						
None	2.641	7.531	0.35	0.727	−12.12 17.402	0.196
Mode of birth						
Forceps assisted delivery	1.413	10.005	0.14	0.888	−18.197 21.023	0.048
Vacuum assisted delivery	−1.9	9.46	−0.20	0.841	−20.442 16.642	0.092
Planned caesarean section	16.416	10.8	1.52	0.133	−4.752 37.584	0.338
Emergency caesarean section	9.686	9.607	1.01	0.317	−9.144 . 28.516	0.208
Birth satisfaction	−0.036	0.608	−0.06	0.953	−1.228 1.156	0.054
Postpartum complication						
None	11.103	8.458	1.31	0.194	−5.475 27.681	0.262
Postpartum length of stay	4.339	2.436	1.78	0.079	−0.436 9.114	0.428
Time elapsed between birth and questionnaire completion	−0.212	0.419	−0.51	0.614	−1.033 0.609	0.124
Overnight stay						
No	−6.91	6.172	−1.12	0.267	−19.007 5.187	0.162
Infant sex						
Boy	−2.381	5.622	−0.42	0.673	−13.4 8.638	0.106
Infant birth weight	−0.956	7.455	−0.13	0.898	−15.568 13.656	0.07
Feeding mode						
Mixed or formula feeding	7.475	8.363	0.89	0.375	−8.916 23.866	0.176
Satisfaction with general informal support	0.005	0.293	0.02	0.988	−0.569 0.579	0.092
Satisfaction with informal informational support	0.296	0.183	1.62	0.11	−0.063 0.655	0.396
Satisfaction with informal instrumental support	−0.107	0.218	−0.49	0.627	−0.534 0.32	0.106
Satisfaction with informal appraisal support	−0.39	0.194	−2.01	0.049	−0.77 −0.01	0.514
Satisfaction with informal emotional support	0.534	0.264	2.02	0.048	0.017 1.051	0.562
**N (63)**	**coefficient**	**Std. error**	**z**	***p***	**95 %CI**	**power**
**Father-infant-bonding**^b^						
Formal social support	0.006	0.004	1.73	0.084	−0.001 0.013	0.53
Age	−0.006	0.019	−0.29	0.771	−0.044 0.033	0.28
Education						
Secondary	0.7	0.297	2.35	0.019	0.12 1.306	0.68
Tertiary	0.164	0.181	0.91	0.363	−0.202 0.519	0.38
Depressive symptoms	0.072	0.024	3.05	0.002	0.025 0.119	0.66
Anxiety symptoms	−0.029	0.028	−1.04	0.299	−0.084 0.027	0.25
Complication during pregnancy						
None	−0.287	0.199	−1.44	0.149	−0.688 0.104	0.38
Mode of birth						
Forceps assisted delivery	−0.41	0.245	−1.67	0.095	−0.894 0.084	0.31
Vacuum assisted delivery	0.136	0.239	0.57	0.569	−0.332 0.62	0.43
Planned caesarean section	0.304	0.3	1.01	0.311	−0.269 0.928	0.276
Emergency caesarean section	−0.216	0.273	−0.79	0.427	−0.743 0.345	0.212
Birth satisfaction	0.019	0.016	1.17	0.24	−0.013 0.051	0.42
Postpartum complication						
None	−0.087	0.221	−0.39	0.694	−0.534 0.345	0.19
Postpartum length of stay	−0.01	0.068	−0.15	0.879	−0.146 0.125	0.18
Time elapsed between birth and questionnaire completion	0.008	0.011	0.69	0.488	−0.014 0.01	0.45
Infant sex						
Boy	0.252	0.138	1.83	0.067	−0.023 0.525	0.5
Infant birth weight	0.424	0.197	2.16	0.031	0.034 0.817	0.45
Feeding mode						
Mixed or formula feeding	0.121	0.21	0.58	0.565	−0.293 0.55	0.23
Satisfaction with general informal support	0.008	0.007	1.07	0.286	−0.007 0.023	0.4
Satisfaction with informal informational support	−0.003	0.006	−0.61	0.545	−0.015 0.008	0.23
Satisfaction with informal instrumental support	0.01	0.006	1.73	0.084	−0.002 0.021	0.62
Satisfaction with informal appraisal support	0.001	0.005	0.20	0.84	−0.01 0.012	0.4
Satisfaction with informal emotional support	−0.018	0.007	−2.53	0.011	−0.033 −0.004	0.64

a Model fit: Adjusted R² = 0.446, F(25, 67) = 2.157, *p* = 0.007.

b Model fit: R² = 0.5770.

**Table 8:** Partner’s self-efficacy and father-infant bonding estimated marginal means per educational level.

	Partners’ self-efficacy			Father-infant bonding		
Education Level	Estimated marginal mean	Standard Error	95 % CI	Estimated marginal mean	Standard Error	95 % CI
Apprenticeship	199	7.31	185 214	0.809	0.176	0.463 1.155
Secondary	164	11.40	141 186	1.508	0.284	0.952 2.064
Tertiary	189	7.14	174 203	0.973	0.136	0.706 1.24

Results are averaged over the levels of infant sex, mode of infant feeding, complication during pregnancy, mode of birth, postpartum complication. Results are given on the log (not the response) scale.

In the hurdle-gamma regression model, 57.7 % of the variance of father-infant bonding was explained (R^2^ = 0.5770). Infant birth weight emerged as the strongest predictor of father-infant bonding difficulties, with higher birth weight associated with more difficulties (β = 0.424, *p* = 0.031). Depressive symptoms were significantly associated with more father-infant bonding difficulties (β = 0.072, *p* < 0.002). Partners with secondary education had more bonding difficulties compared to the “apprenticeship” reference group (β = 0.700, *p* = 0.019). Mode of birth, infant sex, and satisfaction with informal instrumental support showed marginal associations (*p*-values ranging from 0.067 to 0.095). Post-hoc power analysis indicated that these effect sizes were underpowered in our sample (see Table 7). Overall model-wide power across all variables was low, with a mean of 39.6 %.

## 4 Discussion

This study provides new insights into how formal social support provided during the early postpartum hospital stay relates to parenting self-efficacy and bonding in mothers and partners. Overall, parents reported moderate to high levels of perceived formal social support and satisfaction, although satisfaction with the information provided was lower. Formal support was not significantly associated with parenting self-efficacy or parent-infant bonding for either mothers or partners after adjusting for covariates.

Bonding difficulties were relatively low, consistent with previous studies (Stuijfzand et al., 2020; Toivo et al., 2023). Parenting self-efficacy scores were slightly lower than those reported previously (Courtois and Wendland, 2023; Abdollahi et al., 2016). These differences may stem from the timing of the assessment, as most studies measured parenting self-efficacy approximately three months postpartum, whereas our data were collected within the first month, a period of transition to independent caregiving. Evidence suggests parenting self-efficacy declines from the second day after birth to six weeks postpartum (Zheng and Gao, 2023), underscoring the need for continuity of care between hospital and home settings. Healthcare professionals should enhance discharge preparation by ensuring timely post-discharge midwife visits and clear communication channels for parental support (Scroggins et al., 2024). Moreover, the cited studies were conducted in different countries (France, Iran and Canada), which vary in terms of cultural expectations, parental roles, and the organisation of postpartum care. Such contextual differences may also help explain the observed variations in parenting self-efficacy scores.

The majority of mothers and partners reported high satisfaction with formal social support, in line with other European studies (Barimani et al., 2014; Malouf et al., 2019). However, satisfaction with the information received during the postpartum stay was lower. Parents often experience information overload (Loudon et al., 2016; Stoodley et al., 2025) and perceive inconsistent information as unsettling (Schobinger et al., 2022). In McLeish’s study (2021), conflicting advice led some mothers to question professionals’ competencies. Another study in America focusing on mothers’ perspective on supportive professional behaviours and improvement related to the preparation to discharge, found that mothers preferred concise written materials to aid information retention (Scroggins et al., 2024). Providing consistent, prioritised information using diverse formats may therefore improve parental understanding and retention of information (Barimani and Vikström, 2015).

Parents reported moderate to relatively high levels of perceived formal social support responding to their needs; however, wide interquartile ranges indicate that although some parents perceive adequate formal support, others may have perceived it as insufficient or not meeting their needs. Therefore, tailored approaches are essential to ensure all parents receive sufficient support that effectively addresses their needs during the postpartum period (Schobinger et al., 2024).

### 4.1 Relationship between formal social support, parenting self-efficacy, and bonding

Bivariate analyses found a positive association between formal social support and maternal parenting self-efficacy, but not for partners, partially supporting hypothesis one (H1). This association did not remain significant after adjustment for psychosocial, obstetric, and infant-related factors. Formal social support was only measured in one study, which found no association with parenting self-efficacy (Salonen et al., 2009). In our study, the lack of association for partners may reflect their limited presence on the ward and societal norms positioning them as secondary caregivers (Schobinger et al., 2022). Healthcare professionals need to develop strategies to actively involve partners in postpartum care, including encouraging skin-to-skin contact, and offering tailored information and support that acknowledge partners’ specific needs and caregiving role.

No association was found between formal social support received during the postpartum hospital stay and parent-infant bonding, refuting hypothesis two (H2). Only one previous study reported an association; however, formal social support extended beyond the postpartum stay and was assessed using a different instrument (Wells et al., 2024). One possible explanation for the lack of association in our study is the relatively short duration of the postpartum stay, which may not have been sufficient for formal social support to significantly impact parent-infant bonding, as bonding is a gradual process (Nakić Radoš et al., 2024).

In addition to these findings, in our regression model, maternal parenting self-efficacy was positively associated with the satisfaction with general informal support, emphasising the role of informal social support and its association with parenting self-efficacy (Fang et al., 2021). For partners, satisfaction with informal emotional support was positively associated with paternal parenting self-efficacy, suggesting that informal emotional support plays a vital role for partners in developing confidence in parenting. This finding is consistent with previous studies, reporting that first-time fathers often rely on their partner or close relatives for emotional support, which enhances their parenting self-efficacy (Francis Xavier et al., 2024; Saether et al., 2023).

Conversely, partners who reported higher satisfaction with informal appraisal support tended to have lower parenting self-efficacy. One possible explanation is that actively seeking or relying on evaluative feedback may reflect underlying uncertainty in their parenting abilities or stress. This aligns with previous research showing that fathers with lower self-confidence or higher stress exhibit reduced parenting self-efficacy (Fang et al., 2021). Although partners in this study reported being satisfied with such feedback, its effect may sometimes be negative if the support is not delivered appropriately, highlighting the importance of considering not only the availability but also the quality and context of social support (Saether et al., 2023).

The non-significant association between formal social support and maternal parenting self-efficacy in the multivariate analysis may indicate that the relationship is more complex than a direct effect. For example, parenting self-efficacy might be influenced indirectly through other variables such as satisfaction with informal support or mental health status, suggesting possible mediation effects. Because our analyses did not test for mediation explicitly, future research using mediation analysis or structural equation modelling could better clarify these pathways. Overall, our findings support hypothesis three (H3), highlighting the important role of informal social support in parenting self-efficacy, particularly for mothers and, to some extent partners.

### 4.2 Other factors associated with parenting self-efficacy and parent-infant bonding

Beyond social support, other factors were associated with parenting self-efficacy. Partners with secondary education reported significantly lower parenting self-efficacy than those with apprenticeship training. While previous study suggests that higher education may be associated with lower paternal self-efficacy due to elevated expectations (Donithen and Schoppe-Sullivan, 2022), this was not observed among university-educated partners in our study. Their potentially higher standards may have been offset by greater socioeconomic stability or more flexible work–family arrangements. In contrast, secondary education may provide neither the practical orientation of apprenticeship nor the resources of university, leaving partners more vulnerable to reduced self-efficacy. In Switzerland, apprenticeship programmes are particularly practice-oriented, which may foster transferable problem-solving skills and confidence relevant to early caregiving. Nonetheless, this interpretation should be considered exploratory, as a systematic review concluded that the evidence linking education and parenting self-efficacy is mixed (Fang et al., 2021). Future research should explore whether hands-on caregiving experience, occupational self-concept, or peer support networks mediate this effect.

Father-infant bonding was also linked to education: partners with secondary education reported more bonding difficulties compared to those with apprenticeship training. Evidence regarding the association between parental education and bonding remains inconsistent, with some studies reporting greater involvement in infant care, among more educated fathers which could potentially facilitate bonding (Aslan et al., 2017), whereas others report no association (Bieleninik et al., 2021). These findings suggest that education may influence bonding indirectly, potentially through parenting self-efficacy. Indeed, higher parenting self-efficacy has consistently been associated with better parent–infant bonding (Qi et al., 2025; Liu et al., 2022).

In addition, father-infant bonding was associated with newborn weight, with partners having heavier infants reporting more bonding difficulties. Although this contrasts with findings form preterm samples (Taing et al., 2020). In our study with term infants, a different mechanism may be at play. A systematic review has identified birth weight as a factor influencing infant temperament (Takegata et al., 2021). Indeed, heavier infants may be more likely to exhibit a difficult temperament (Niegel et al., 2007), which may challenge fathers’ parenting self-efficacy and reduce their confidence in meeting their child’s needs, thereby affecting bonding (Qi et al., 2025). This interpretation remains speculative and warrants further investigation.

Mother-infant bonding was associated with maternal age and infant feeding mode. Older maternal age was linked to more bonding difficulties, consistent with findings from a systematic review reporting negative associations between maternal age and bonding quality (Tichelman et al., 2019). However, a more recent study found that younger maternal age predicted poorer bonding (Doyle et al., 2023). This suggests the relationship between maternal age and bonding is complex and may depend on other contextual factors. Our results may be explained by older mothers experiencing more fatigue, slower recovery, or greater competing responsibilities, which could reduce opportunities for early bonding with their infant (La Rosa et al., 2025). Mothers who did not exclusively breastfeed also reported more bonding difficulties. While evidence on the relationship between breastfeeding and bonding remains complex and debated (Bicking Kinsey and Hupcey, 2013; Doyle et al., 2023), exclusive breastfeeding may facilitate bonding (Nakić Radoš et al., 2024), through increased physical contact (skin-to-skin), inducing oxytocin release, a hormone involved in bonding (Modak et al., 2023). Breastfeeding may also facilitate early parent–infant interactions, thereby enhancing maternal self-efficacy, which in turn is positively related to bonding (Modak et al., 2023; Qi et al., 2025). Conversely, mothers who do not exclusively breastfeed may experience feelings of inadequacy or guilt, particularly if they intended to breastfeed, which can negatively influence their perception of self-efficacy (Russell et al., 2021) and, consequently the quality of their bond.

Finally depressive symptoms were significantly associated with both lower parenting self-efficacy and parent-infant bonding, consistent with previous research (Fang et al., 2021; Albanese et al., 2019). Depression symptoms may impair emotional availability and parental engagement (Döblin et al., 2023; Ngai and Lam, 2023), highlighting the importance of early identification of parental distress. Training healthcare professionals to recognise signs of parental distress and providing appropriate emotional support or referrals to mental health services should be prioritised.

Overall, our findings support hypothesis three (H3), highlighting the important role of informal social support in enhancing parenting self-efficacy, especially among mothers and, to some extent, partners. Additionally, several other factors—such as education, infant feeding mode and depressive symptoms—significantly influence parenting self-efficacy and parent-infant bonding. These results emphasise the complex interplay between these variables and call for tailored formal support that addresses parents’ specific needs during the postpartum period.

### 4.3 Strengths and limitations

Strengths include the inclusion of both parents and the use of validated questionnaires. However, several limitations should be noted. Fewer partners participated than mothers, and post-hoc power analyses indicated that some regression estimates were underpowered, particularly in partners’ analyses, potentially limiting the detection of associations. Nevertheless, several significant associations were identified between informal support, depressive symptoms, and parenting self-efficacy and bonding, highlighting meaningful relationships despite these limitations. Additionally, most participants were Swiss, highly educated, living in couples, and gave birth in a university hospital, which may limit generalisability to more diverse populations. Potential non-independence between mothers and partners from the same couple could not be accounted for, as pairing information was unavailable. Inherent to cross-sectional design, causal inferences cannot be drawn. Future studies with larger samples of fathers/partners and prospective designs are needed to explore these relationships. Another limitation relates to the analytical approach. Although multivariate regressions were conducted, mediation effects were not examined. Future studies should apply mediation analyses or structural equation modelling to disentangle these complex relationships.

## 5 Conclusions

This study provides new insights into the association between formal support received during the postpartum stay, parenting self-efficacy, and parent-infant bonding, showing that formal social support was not related to these outcomes, even after accounting for confounding factors. Satisfaction with information received from healthcare professionals was lower than with other types of support. Factors such as depression symptoms and satisfaction with informal social support emerged as key correlates of parenting self-efficacy and parent-infant bonding, emphasising the importance of mental health and informal networks in early parenthood.

## Ethics approval and consent to participate

This study was approved by the Human Research and Ethics Committee of the Canton of Vaud, Switzerland (Project No 2021-00762). Written informed consent was not necessary, as the study was categorised as anonymous, and parents were deemed to consent by completing the online survey.

## Consent for publication

Not applicable.

## Availability of data and materials

The data collected for this study cannot be shared at this time as further analysis is still ongoing, particularly related to the validation of a tool that is currently in preparation. Once the analysis is completed, the data will be deposited in a relevant data repository and made available for citation and linking in the article.

## Funding

This research did not receive any specific grant from funding agencies in the public, commercial, or not-for-profit sectors.

## CRediT authorship contribution statement

**Elisabeth Schobinger:** Writing – review & editing, Writing – original draft, Methodology, Investigation, Formal analysis, Data curation, Conceptualization. **Alain Lacroix:** Writing – review & editing, Supervision, Software, Formal analysis, Data curation. **Anne-Sylvie Ramelet:** Writing – review & editing, Validation, Supervision, Methodology, Conceptualization. **Antje Horsch:** Writing – review & editing, Validation, Supervision, Conceptualization.

## Declaration of competing interest

The authors declare that they have no known competing financial interests or personal relationships that could have appeared to influence the work reported in this paper.

## References

[bib0001] Abdollahi F., Agajani-Delavar M., Zarghami M., Lye M. (2016). Postpartum mental health in first-time mothers: a cohort study. Iran. J. Psychiatry Behav. Sci..

[bib0002] Albanese A.M., Russo G.R., Geller P.A. (2019). The role of parental self-efficacy in parent and child well-being: a systematic review of associated outcomes. Child Care Health Dev..

[bib0003] Aslan E., Erturk S., Demir H., Aksoy O. (2017). Fathers' attachment status to their infants. Int. J. Caring Sci..

[bib0004] Ayers S., Crawley R., Webb R., Button S., Thornton A., The H.C.G., Smith H., Bradley R., Lee S., Moore D., Field A., Eagle A., Gyte G. (2019). What are women stressed about after birth?. Birth.

[bib0005] Azmoude E., Jafarnejade F., Mazloum S.R. (2015). The predictors for maternal self-efficacy in early parenthood. JMRH,.

[bib0006] Baldwin S., Malone M., Sandall J., Bick D. (2019). A qualitative exploratory study of UK first-time fathers’ experiences, mental health and wellbeing needs during their transition to fatherhood. BMJ Open.

[bib0007] Bandura A. (1997).

[bib0008] Barimani M., Oxelmark L., Johansson S.-E., Langius-Eklöf A., Hylander I. (2014). Professional support and emergency visits during the first 2 weeks postpartum. Scand. J. Caring Sci..

[bib0009] Barimani M., Vikström A. (2015). Successful early postpartum support linked to management, informational, and relational continuity. Midwifery.

[bib0010] Beshir M., Tilahun T., Hordofa D.F., Abera G., Tesfaye W., Daba K.T., Workineh N., Woldeyesus S.N., Debela T.F., Yesuf E.A. (2022). Caregiver satisfaction and its associated factors in pediatric wards of Jimma University Medical Center, Southwest Ethiopia. BMC Health Serv. Res..

[bib0011] Bicking Kinsey C., Hupcey J.E. (2013). State of the science of maternal-infant bonding: a principle-based concept analysis. Midwifery.

[bib0012] Bieleninik Ł., Lutkiewicz K., Jurek P., Bidzan M. (2021). Paternal postpartum bonding and its predictors in the early postpartum period: cross-sectional study in a Polish cohort. Front. Psychol..

[bib0013] Bienfait M., Haquet A., Maury M., Faillie J.-L., Combes C., Cambonie G. (2017). Traduction française de l’autoquestionnaire MIBS (mother to infant bonding scale) et validation comme évaluation du lien mère-nouveau-né en maternité. Devenir.

[bib0014] Boini, S. & Langevin, V. 2020;. Hospital anxiety and depression scale (HADS) références en santé au travail**,** 123–7.

[bib0015] Brand R.J., Gartland C.A., Koo G., Mcmahon J.E., Hicks J.M., Al-Khayyat R., Jaatinen M.M. (2025). Perceived autonomy during childbirth predicts mothers’ parental self-efficacy: a prospective cohort study. J. Health Psychol..

[bib0016] Cirino N.H., Knapp J.M. (2019). Perinatal Posttraumatic Stress Disorder: a review of risk factors, diagnosis, and treatment. Obstet. Gynecol. Surv..

[bib0017] Courtois E., Wendland J. (2023). L’accouchement vécu négativement par les pères est associé à une augmentation du risque de dépression paternelle en post-partum. Gynecol. Obstet. Fertil. Senol..

[bib0018] De Montigny F. (2002).

[bib0019] De Montigny F., Lacharité C. (2002). Perceptions des pères et des mères primipares à l’égard des moments critiques des 72 premières heures postnatales. Rev. Québ. Psychol..

[bib0020] De Montigny F., Lacharité C. (2005). Perceived parental efficacy: concept analysis. J. Adv. Nurs..

[bib0021] De Waal N., Van Den Heuvel M.I., Nyklíček I., Pop V.J.M., Boekhorst M.G.B.M (2025). Paternal bonding in pregnancy and early parenthood: a qualitative study in first-time fathers. J. Reprod. Infant Psychol..

[bib0022] Devita S., Lacroix A., Avignon V., Rattaz V., Deforges C., Horsch A. (2026). Validity and reliability of the French version of the birth satisfaction scale-revised (F-BSS-R). J. Reprod. Infant Psychol..

[bib0023] Döblin S., Seefeld L., Weise V., Kopp M., Knappe S., Asselmann E., Martini J., Garthus-Niegel S. (2023). The impact of mode of delivery on parent-infant-bonding and the mediating role of birth experience: a comparison of mothers and fathers within the longitudinal cohort study DREAM. BMC Pregnancy Childbirth.

[bib0024] Donithen R., Schoppe-Sullivan S. (2022). Correlates and predictors of parenting self-efficacy in new fathers. J. Fam. Psychol..

[bib0025] Doyle F.L., Dickson S.J., Eapen V., Frick P.J., Kimonis E.R., Hawes D.J., Moul C., Richmond J.L., Mehta D., Dadds M.R. (2023). Towards preventative psychiatry: concurrent and longitudinal predictors of postnatal maternal-infant bonding. Child Psychiatry Hum. Dev..

[bib0026] Eddy B., Poll V., Whiting J., Clevesy M. (2019). Forgotten fathers: postpartum depression in men. J. Fam. Issues.

[bib0027] Ekström-Bergström A., Thorstensson S., Bäckström C. (2022). The concept, importance and values of support during childbearing and breastfeeding – a discourse paper. Nurs. Open.

[bib0028] Falk M., Nelson M., Blomberg M. (2019). The impact of obstetric interventions and complications on women’s satisfaction with childbirth a population based cohort study including 16,000 women. BMC Pregnancy Childbirth.

[bib0029] Fang Y., Boelens M., Windhorst D.A., Raat H., Van Grieken A. (2021). Factors associated with parenting self-efficacy: a systematic review. J. Adv. Nurs..

[bib0030] Fierloos I.N., Windhorst D.A., Fang Y., Hosman C.M.H., Jonkman H., Crone M.R., Jansen W., Raat H. (2023). The association between perceived social support and parenting self-efficacy among parents of children aged 0–8 years. BMC Public Health.

[bib0031] Finlayson K., Crossland N., Bonet M., Downe S. (2020). What matters to women in the postnatal period: a meta-synthesis of qualitative studies. PLoS ONE.

[bib0032] Francis Xavier J., Khanlou N., Nielsen L.S., Moradian S. (2024). Enhancing paternal support: a concept analysis of social support for first-time fathers. Nurs. Forum.

[bib0033] Hodgson S., Painter J., Kilby L., Hirst J. (2021). The experiences of first-time fathers in perinatal services: present but invisible. Healthcare (Basel).

[bib0034] Hollins Martin C.J., Martin C.R. (2014). Development and psychometric properties of the birth satisfaction scale-revised (BSS-R). Midwifery.

[bib0035] Horsch A., Jacobs I., Gilbert L., Favrod C., Schneider J., Morisod Harari M., Bickle Graz M. (2017). Impact of perinatal asphyxia on parental mental health and bonding with the infant: a questionnaire survey of Swiss parents. BMJ Paediatr. Open.

[bib0036] Kothari A., Bruxner G., Callaway L., Dulhunty J.M. (2022). It’s a lot of pain you’ve got to hide”: a qualitative study of the journey of fathers facing traumatic pregnancy and childbirth. BMC Pregnancy Childbirth.

[bib0037] La Rosa V.L., Alparone D., Commodari E. (2025). Psychological and social factors influencing mother-child bonding in the first year after birth: a model for promoting infant and maternal well-being. Front. Psychol..

[bib0038] Langford C.P.H., Bowsher J., Maloney J.P., Lillis P.P. (1997). Social support: a conceptual analysis. J. Adv. Nurs..

[bib0039] Leahy-Warren P. (2014).

[bib0040] Leahy-Warren P., Mccarthy G., Corcoran P. (2012). First-time mothers: social support, maternal parental self-efficacy and postnatal depression. J. Clin. Nurs..

[bib0041] Leahy-Warren P., Mulcahy H., Lehane E. (2019). The development and psychometric testing of the perinatal infant care social support (PICSS) instrument. J. Psychoso. Res..

[bib0042] Liu C.H., Hyun S., Mittal L., Erdei C. (2022). Psychological risks to mother–infant bonding during the COVID-19 pandemic. Pediatr. Res..

[bib0043] Liyana Amin N.A., Tam W.W.S., Shorey S. (2018). Enhancing first-time parents' self-efficacy: a systematic review and meta-analysis of universal parent education interventions' efficacy. Int. J. Nurs. Stud..

[bib0044] Loudon K., Buchanan S., Ruthven I. (2016). The everyday life information seeking behaviours of first-time mothers. J. Document..

[bib0045] Malouf R., Henderson J., Alderdice F. (2019). Expectations and experiences of hospital postnatal care in the UK: a systematic review of quantitative and qualitative studies. BMJ Open.

[bib0046] Martin R.C.B., Brock R.L. (2023). The importance of high-quality partner support for reducing stress during pregnancy and postpartum bonding impairments. Arch. Womens Ment. Health.

[bib0047] Mcleish J., Harvey M., Redshaw M., Alderdice F. (2021). A qualitative study of first time mothers’ experiences of postnatal social support from health professionals in England. Women Birth.

[bib0048] Modak A., Ronghe V., Gomase K.P. (2023). The psychological benefits of breastfeeding: fostering maternal well-being and child development. Cureus.

[bib0049] Nakić Radoš S., Hairston I., Handelzalts J.E. (2024). The concept analysis of parent-infant bonding during pregnancy and infancy: a systematic review and meta-synthesis. J. Reprod. Infant Psychol..

[bib0050] Ngai F.-W., Lam W. (2023). Predictors of parent-infant bonding among postpartum Chinese mothers and fathers. J. Midwifery Womens Health.

[bib0051] Niegel S., Ystrom E., Vollrath M.E. (2007). Is difficult temperament related to overweight and rapid early weight gain in infants? A prospective cohort study. J. Dev. Behav. Pediatr..

[bib0052] Office Fédéral De La Statistique (2019).

[bib0053] Qi W., Wei Z., Lv H., Zhao J., Hu Y., Wang Y., Guo Q., Hu J. (2025). Postpartum depression and maternal-infant bonding: the mediating role of mentalizing and parenting self-efficacy. BMC Pregnancy Childbirth.

[bib0054] R Core Team (2024).

[bib0055] Razurel C., Kaiser B., Antonietti J.-P., Epiney M., Sellenet C. (2017). Relationship between perceived perinatal stress and depressive symptoms, anxiety, and parental self-efficacy in primiparous mothers and the role of social support. Women Health.

[bib0056] Russell P.S., Birtel M.D., Smith D.M., Hart K., Newman R. (2021). Infant feeding and internalized stigma: the role of guilt and shame. J. Appl. Soc. Psychol..

[bib0057] Saether K.M., Berg R.C., Fagerlund B.H., Glavin K., Jøranson N. (2023). First-time parents' experiences related to parental self-efficacy: a scoping review. Res. Nurs. Health.

[bib0058] Salonen A.H., Kaunonen M., Astedt-Kurki P., Järvenpää A.L., Isoaho H., Tarkka M.T. (2009). Parenting self-efficacy after childbirth. J. Adv. Nurs..

[bib0059] Samdan G., Reinelt T., Kiel N., Mathes B., Pauen S. (2022). Maternal self-efficacy development from pregnancy to 3 months after birth. Infant Ment. Health J..

[bib0060] Schobinger E., Horsch A., Ramelet A.-S. (2025). Delphi study on first-time parents perceptions of professional support in the early postpartum period. Midwifery.

[bib0082] Schobinger E., Lacroix A., Horsch A., Ramelet A.-S. (2025). Development and measurement properties of the formal social support scale postpartum (FOCUS-postpartum) for first-time mothers. BMC Pregnancy Childbirth.

[bib0061] Schobinger E., Stuijfzand S., Horsch A. (2020). Acute and post-traumatic stress disorder symptoms in mothers and fathers following childbirth: a prospective cohort study. Front. Psychiatry.

[bib0062] Schobinger E., Vanetti M., Ramelet A.-S., Horsch A. (2022). Social support needs of first-time parents in the early-postpartum period: a qualitative study. Front. Psychiatry.

[bib0063] Schobinger E., Vanetti M., Ramelet A.-S., Horsch A. (2024). First-time parents’ perception of midwives’ and other healthcare professionals’ support behaviours: a qualitative study. Midwifery.

[bib0064] Scroggins J.K., Gibson A.N., Stuebe A.M., Sheffield-Abdullah K.M., Tully K.P. (2024). Postpartum hospital discharge: birthing parent perspectives on supportive practices and areas for improvement. J. Perinat. Neonatal Nurs..

[bib0065] Shorey S., Chan S.W.-C., Chong Y.S., He H.-G. (2014). Maternal parental self-efficacy in newborn care and social support needs in Singapore: a correlational study. J. Clin. Nurs..

[bib0066] Stoodley C., Mckellar L., Fereday J., Ziaian T., Steen M., Gwilt I. (2025). Exploring mothers' perspectives on the early mother-infant relationship to inform midwifery practice: a qualitative study. Midwifery.

[bib0067] Stuijfzand S., Garthus-Niegel S., Horsch A. (2020). Parental birth-related PTSD symptoms and bonding in the early postpartum period: a prospective population-based cohort study. Front. Psychiatry.

[bib0068] Taing R., Galescu O., Noble L., Hand I.L. (2020). Factors influencing paternal attachment among preterm infants in an urban neonatal intensive care unit. Cureus.

[bib0069] Takegata M., Matsunaga A., Ohashi Y., Toizumi M., Yoshida L.M., Kitamura T. (2021). Prenatal and intrapartum factors associated with infant temperament: a systematic review. Front. Psychiatry.

[bib0070] Taylor A., Atkins R., Kumar R., Adams D., Glover V. (2005). A new mother-to-infant bonding scale: links with early maternal mood. Arch. Womens Ment. Health.

[bib0071] Tichelman E., Westerneng M., Witteveen A.B., Van Baar A.L., Van Der Horst H.E., De Jonge A., Berger M.Y., Schellevis F.G., Burger H., Peters L.L. (2019). Correlates of prenatal and postnatal mother-to-infant bonding quality: a systematic review. PLoS One.

[bib0072] Toivo J., Tulivuo N., Kanzaki M., Koivisto A.-M., Kylmä J., Paavilainen E. (2023). First-time parents’ bonding with their baby: a longitudinal study on Finnish parents during the First eight months of parenthood. Children.

[bib0073] Van Buuren S. (2018).

[bib0074] Vogel R., Mcgraw C., Orlando A., Bourg P., Dreiman C., Peck L., Tanner A., Lynch N., Bar-Or D. (2019). Examining satisfaction of older adult patients and their caregivers following traumatic injury: a cross-sectional study of three level I trauma centres. BMJ Open.

[bib0075] Voorhees H.L. (2023). The International Encyclopedia of Health Communication.

[bib0076] Voutilainen A., Pitkäaho T., Kvist T., Vehviläinen-Julkunen K. (2016). How to ask about patient satisfaction? The visual analogue scale is less vulnerable to confounding factors and ceiling effect than a symmetric Likert scale. J. Adv. Nurs..

[bib0077] Wells M.B., Giannotti M., Aronson O. (2024). Partner and professional support are associated with father-infant bonding: a cross-sectional analysis of mothers, midwives, and child health nurses’ influence on primiparous and multiparous fathers of infants in Sweden. Midwifery.

[bib0078] White I.R., Royston P., Wood A.M. (2011). Multiple imputation using chained equations: issues and guidance for practice. Stat. Med..

[bib0079] Wild D., Grove A., Martin M., Eremenco S., Mcelroy S., Verjee-Lorenz A., Erikson P. (2005). Principles of good practice for the translation and cultural adaptation process for patient-reported outcomes (PRO) measures: report of the ISPOR Task Force for Translation and Cultural Adaptation. Value Health.

[bib0080] World Health Organisation 2010. WHO Technical Consultation On Postpartum and Postnatal Care [Online]. Geneva: World Health Organization,. 26269861

[bib0081] World Health Organisation 2022 WHO Recommendations On Maternal and Newborn Care For a Positive Postnatal Experience [On Line]. Geneva. 35467813

[bib0083] Zheng J., Gao L.-L. (2023). Parenting self-efficacy and social support among parents in mainland China across the first six months postpartum: a prospective cohort study. Midwifery.

[bib0084] Zheng X., Morrell J., Watts K. (2018). A quantitative longitudinal study to explore factors which influence maternal self-efficacy among Chinese primiparous women during the initial postpartum period. Midwifery.

[bib0085] Zigmond A.S., Snaith R.P. (1983). The hospital anxiety and depression scale. Acta Psychiatr. Scand..

